# Generation of Priority Research Questions to Inform Conservation Policy and Management at a National Level

**DOI:** 10.1111/j.1523-1739.2010.01625.x

**Published:** 2011-06

**Authors:** Murray A Rudd, Karen F Beazley, Steven J Cooke, Erica Fleishman, Daniel E Lane, Michael B Mascia, Robin Roth, Gary Tabor, Jiselle A Bakker, Teresa Bellefontaine, Dominique Berteaux, Bernard Cantin, Keith G Chaulk, Kathryn Cunningham, Rod Dobell, Eleanor Fast, Nadia Ferrara, C Scott Findlay, Lars K Hallstrom, Thomas Hammond, Luise Hermanutz, Jeffrey A Hutchings, Kathryn E Lindsay, Tim J Marta, Vivian M Nguyen, Greg Northey, Kent Prior, Saudiel Ramirez-Sanchez, Jake Rice, Darren J H Sleep, Nora D Szabo, Geneviève Trottier, Jean-Patrick Toussaint, Jean-Philippe Veilleux

**Affiliations:** 1 Environment Department, University of YorkHeslington, York YO10 5DD, United Kingdom email murray.rudd@york.ac.uk; 2 School for Resource and Environmental Studies, Dalhousie UniversityHalifax, NS B3H 3J5, Canada; 3 Department of Biology and Institute of Environmental Science, Carleton UniversityOttawa, ON K1S 5B6, Canada; 4 Bren School of Environmental Science & Management, University of CaliforniaSanta Barbara, CA 93106-3060, U.S.A.; 5 TelferSchool of Management, University of OttawaOttawa, ON K1N 6N5, Canada; 6 Conservation Science ProgramWorld Wildlife Fund, 1250 24th Street NW, Washington, D.C. 20009, U.S.A.; 7 Department of Geography, York UniversityToronto, ON M3J 1P3, Canada; 8 Center for Large Landscape Conservation, Yellowstone to Yukon Conservation InitiativeP.O. Box 1587, Bozeman, MT 59771, U.S.A.; 9 Department of Environmental Sciences, University of OttawaOttawa, ON K1N 6N5, Canada; 10 Policy Research Initiative56 Sparks Street, Ottawa, ON K1P 5A9, Canada; 11 Chaire de recherche du Canada en conservation des écosystèmes nordiques and Centre d'études nordiques, Université du Québec à RimouskiRimouski, PQ G5L 3A1, Canada; 12 Policy Research Initiative56 Sparks Street, Ottawa, ON K1P 5A9, Canada; 13 Labrador Institute, Memorial University of NewfoundlandHappy Valley – GooseBay, NL A0P 1E0, Canada; 14 Ocean Management Research NetworkOttawa, ON K1N 6N5, Canada; 15 Centre for Global Studies, University of VictoriaVictoria, BC V8W 2Y2, Canada; 16 Council of Canadian Academies180 Elgin St., Suite 1401, Ottawa, ON K2P 2K3, Canada; 17 Sustainable Development Division, Indian and Northern Affairs CanadaOttawa, ON K1A 0H4, Canada; 18 Department of Biology and Institute of the Environment, University of OttawaOttawa, ON K1N 6N5, Canada; 19 Alberta Centre for Sustainable Rural Communities, University of AlbertaCamrose, AB T4V 2R3, Canada; 20 Commission for Environmental Cooperation393 St-Jacques Street West Suite 200, Montreal, PQ H2Y 1N9, Canada; 21 Department of Biology, Memorial University of NewfoundlandSt. John's, NL A1B X Canada; 22 Department of Biology, Dalhousie UniversityHalifax, NS B3H 4J1, Canada; 23 Wildlife and Landscape Science DirectorateScience and Technology Branch, Environment Canada, Ottawa, ON K1A 0H3 Canada; 24 Research Branch, Agriculture and Agri-FoodCanadaOttawa, ON K1A 0C5, Canada; 25 Department of Biology, Carleton UniversityOttawa, ON K1S 5B6, Canada; 26 Canadian Federation of Agriculture21 Florence Street, Ottawa, ON K2P 0W6, Canada; 27 Ecological Integrity Branch, Parks Canada AgencyOttawa, ON, Canada; 28 Oceans Directorate, Oceans Policy and Planning Branch, Fisheries and Oceans CanadaOttawa, ON K1A 0E6, Canada; 29 Department of Fisheries and Oceans CanadaOttawa, ON K1A 0E6, Canada; 30 National Council for Air and Stream ImprovementMontreal, PQ H3B 3K5, Canada; 31 Department of Biology, University of OttawaOttawa, ON K1N 6N5, Canada; 32 Canadian Food Inspection AgencyOttawa, ON K1A 0Y9, Canada; 33 Institut National de la Recherche Scientifique – Institut Armand-Frappier, Université du QuébecLaval, & David Suzuki Foundation, Montreal, PQ H3B 1A7, Canada; 34 Policy Research Initiative56 Sparks Street, Ottawa, ON K1P 5A9, Canada

**Keywords:** collaboration, evidence-based policy, research impact, research priorities, science advice, asesoría científica, colaboración, impacto de la investigación, políticas basadas en evidencia, prioridades de investigación

## Abstract

Integrating knowledge from across the natural and social sciences is necessary to effectively address societal tradeoffs between human use of biological diversity and its preservation. Collaborative processes can change the ways decision makers think about scientific evidence, enhance levels of mutual trust and credibility, and advance the conservation policy discourse. Canada has responsibility for a large fraction of some major ecosystems, such as boreal forests, Arctic tundra, wetlands, and temperate and Arctic oceans. Stressors to biological diversity within these ecosystems arise from activities of the country's resource-based economy, as well as external drivers of environmental change. Effective management is complicated by incongruence between ecological and political boundaries and conflicting perspectives on social and economic goals. Many knowledge gaps about stressors and their management might be reduced through targeted, timely research. We identify 40 questions that, if addressed or answered, would advance research that has a high probability of supporting development of effective policies and management strategies for species, ecosystems, and ecological processes in Canada. A total of 396 candidate questions drawn from natural and social science disciplines were contributed by individuals with diverse organizational affiliations. These were collaboratively winnowed to 40 by our team of collaborators. The questions emphasize understanding ecosystems, the effects and mitigation of climate change, coordinating governance and management efforts across multiple jurisdictions, and examining relations between conservation policy and the social and economic well-being of Aboriginal peoples. The questions we identified provide potential links between evidence from the conservation sciences and formulation of policies for conservation and resource management. Our collaborative process of communication and engagement between scientists and decision makers for generating and prioritizing research questions at a national level could be a model for similar efforts beyond Canada.

Generación de Preguntas de Investigación Prioritarias para Informar a las Políticas y Gestión de la Conservación a Nivel Nacional

## Abstract (sp)

**Resumen:**

La integración del conocimiento proveniente de las ciencias sociales y naturales es necesaria para atender efectivamente los pros y contras sociales de la utilización de la biodiversidad por humanos y su preservación. Los procesos de colaboración pueden cambiar la forma en que los tomadores de decisiones piensan sobre la evidencia científica, incrementar los niveles de confianza y credibilidad mutua y avanzar en el discurso político de la conservación. Canadá tiene responsabilidad por una gran proporción de algunos de los principales ecosistemas, como bosques boreales, tundra Ártica, humedales y océanos templados y Ártico. Factores estresantes para la diversidad biológica en esos ecosistemas se originan de actividades de la economía del país basada en recursos, así como de factores externos causantes del cambio ambiental. El manejo efectivo es complicado por la incongruencia entre los límites ecológicos y políticos y por perspectivas conflictivas de las metas sociales y económicas. Muchos vacíos de información sobre los factores estresantes y su manejo podrían reducirse por medio de investigaciones dirigidas y oportunas. Identificamos 40 preguntas que, sí son atendidas o respondidas, harían avanzar a la investigación que tiene una alta probabilidad de soportar el desarrollo de políticas y estrategias de manejo efectivas para especies, ecosistemas y procesos ecológicos en Canadá. Un total de 396 preguntas propuestas, derivadas de disciplinas naturales y sociales, fueron aportadas por individuos de afiliaciones organizacionales diferentes y fueron reducidas a 40 por nuestro equipo de colaboradores. Estas preguntas enfatizan el entendimiento de los ecosistemas, los efectos y mitigación del cambio climático, la coordinación de esfuerzos de gobernanza y manejo en jurisdicciones múltiples y el examen de las relaciones entre las políticas de conservación y el bienestar social y económico de los pueblos aborígenes. Las preguntas que identificamos proporcionan posibles vínculos entre la evidencia aportada por las ciencias de la conservación y la formulación de políticas para la conservación y el manejo de los recursos. Nuestro proceso colaborativo de comunicación y compromiso entre científicos y tomadores de decisiones para generar y priorizar preguntas de investigación a nivel nacional podría ser un modelo para esfuerzos similares fuera de Canadá.

## Introduction

Although we recognize the benefits of ongoing curiosity-driven research, we believe there is clear value in aligning research in the natural and social sciences more closely with the information needs of decision makers. Scientific research can inform decisions regarding domestic environmental protection and enhance a country's ability to contribute to international solutions to global environmental challenges (Government of Canada 2000). In Canada, where social and governance priorities are evolving and governance capacity is relatively high, the process of generating priority research questions to inform conservation policy and management may provide general insights into the demand for research in conservation science and the science–policy interface.

Targeted research can have instrumental effects (Rigby 2005; Nutley et al. 2007), which directly influence policy or practice in specific ways, and conceptual effects (Weiss 1978), which contribute knowledge and understanding that informs decision making over time. Targeted research can help avoid policy failure during the policy formulation stage by improving understanding of causal linkages between stressors, interventions, and effects, and during implementation, when rigorous examination of evidence can help minimize the probability of adverse consequences of individual policies (Bobrow and Dryzek 1987; Dobell 2002; Howlett 2009). As in other fields (Nutley et al. 2007; Howlett 2009), evidence-based policymaking is increasingly being advocated and used in conservation (Pullin et al. 2009).

Recent projects have been undertaken at the global (Sutherland et al. 2009) and national (Sutherland et al. 2006; Morton et al. 2009) levels and on particular topics (e.g., Pretty et al. 2010) to identify conservation research priorities. Prior national efforts focused strictly on ecological questions of relevance for conservation policy. Our work differed from those exercises by aiming to identify both natural and social science research questions that, if addressed or answered, would provide the basis for development and implementation of effective policies and management strategies for species, ecosystems, and ecological processes at the national level. An exercise in the United States (Fleishman et al., unpublished data) was run in parallel with our project, which allowed for some comparison and contrast of conservation priorities across North America.

Historically, natural resource industries were the basis of Canada's economy. Today the contribution of nature to the well-being of individuals, communities, and society is recognized as much greater than simply a source of raw materials. Conservation of biological diversity or *biodiversity*, defined here as “the variety of genes, species and ecosystems and the ecological processes that allow them to evolve and adapt to a changing world” (Environment Canada 2006), is emphasized in legislation and policy by federal, provincial, territorial, local, and Aboriginal governments. This reflects the view that conservation, as well as the sustainable use of natural resources, is an important priority for Canadians (Leonard et al. 2007; Rudd 2009).

## Methods

### Participants

A core team of eight organizers (the eight lead authors) designed and implemented the project. A larger group—who participated in solicitation and synthesis of questions—included policy advisors and decision makers in governments and nongovernmental organizations (e.g., national environmental advocacy and industry organizations) and academics with expertise in conservation science. Of 57 invitees to that group, 43 responded and 28 accepted. The final group of participants was drawn from federal government agencies with national responsibilities (*n*= 10), environmental nongovernmental organizations (*n*= 3), industry associations (*n*= 2), policy analysis or advisory organizations (*n*= 6), and academia (*n*= 7). The core team of eight organizers and four student assistants brought the total number of participants to 40. Twenty-eight of the participants attended a workshop in Ottawa, the national capitol, in April 2010. Although provincial and municipal governments also have substantive, often paramount, responsibilities for ecosystem management, involving participants from a full range of regional government organizations was not feasible logistically. Academic participants were drawn from across the country and many team members had extensive regional policy experience.

### Solicitation of Questions

Participants solicited questions from within their organizations, from colleagues, and via public forums (e.g., discipline-specific email lists and social-networking sites). Questions were sought from policymakers and their advisors in government, policy specialists in the nongovernmental and private sectors, Aboriginal leaders and science advisors, researchers in the natural and social sciences, and the philanthropic community. We used a bilingual (English and French) website established for this exercise to collect questions over 5 weeks.

Instructions for individuals who wished to submit questions were similar to those for other priority-setting exercises (Sutherland et al. 2010), although policy applications were stressed. Individuals were asked, “What research question, if answered, would substantially advance the development of effective policies and management strategies for species, ecosystems, and ecological processes in Canada?” Aspirational criteria for questions were that they (1) be answerable through a realistic research design, (2) be answerable on the basis of facts rather than value judgments, (3) be of a spatial and temporal scale that could be addressed realistically by a research team or program, (4) not be answerable simply with a *yes*, *no*, or *it depends* response, (5) contain a subject of the intervention, an intervention, and a measurable effect if related to interventions and effects, and (6) increase the effectiveness of policy about, and management of, resource use and biodiversity in the face of environmental stressors if answered.

A total of 396 questions were received in 271 submissions. Questions were submitted by individuals with diverse organizational affiliations (68 and 38 from federal and provincial governments respectively, 75 from academia, 33 from nongovernmental organizations, and 57 from other organizations). Over 62% (159 of 255) of individuals who voluntarily provided background professional information had more than 10 years experience in natural resource management. Core team members culled candidate questions that failed to meet the aspirational criteria and combined and refined questions to avoid substantial redundancies. This process reduced the list to 242 questions for participants to consider in the workshop. All questions are included in the Supporting Information.

### Selecting the Top 40

At the workshop, participants selected the top 40 questions from those submitted through a process developed for similar priority-setting exercises in the United Kingdom and United States (Sutherland et al. 2010; Fleishman et al., unpublished data). The workshop opened with a plenary session that included participant introductions, an overview of how participants were selected, highlights from recent exercises, an explanation of the policy emphasis of this exercise, and a review of workshop processes and ground rules. The balance of the day was spent in three breakout sessions (75–90 minutes each) composed of three concurrent groups per session. Each breakout group discussed one loosely themed set of 25–30 candidate questions, narrowing the set to four candidates for the top 40 and two alternates. Participants in breakout groups sometimes combined candidate questions, developed alternate questions that captured key ideas from candidate questions, and referred to the original list of 396 questions. The 54 questions that resulted from the first day (nine breakout groups, six questions each) were discussed during a plenary session on the second day, during which the top 40 were selected by consensus.

After the workshop, the core team and participants, including those individuals unable to attend the workshop in person, refined the questions by consensus-based email dialogue. During the refinement phase, it became apparent that some questions were quite similar. Combining three pairs of questions allowed us to include other candidates that were not on the initial list of 40. Participation of scientists helped ensure that questions were sufficiently narrow to form the basis of a research program. Participation of policymakers helped ensure that the final questions were relevant.

## Results

We grouped the top 40 questions into themes: ecosystem structure and function, land cover and habitats, populations and species, resource-based industries, parks and protected areas, environmental change, environmental values, economic benefits and costs, individual and community well-being, adaptive management, and policy and governance. The organization of these themes was drawn from the adaptive-management logic framework outlined in Rudd (2004). In this framework, ecosystems provide resource flows that, when combined with other flows from other assets (e.g., financial and human capital), are used to undertake activities. Those activities have outputs and outcomes that, combined with external driving forces, affect ecosystem structure and function. Human values act as a filter through which society identifies threats to its well-being. Policy and management interventions can be used to address threats and more closely align the interests and behavior of individuals with the interests of society.

### Ecosystem Structure and Function

Ecosystem attributes that provide benefits to humans (i.e., ecosystem services) are increasingly a focus of biodiversity policy (MEA 2005; Fisher et al. 2009; TEEB 2009). The alteration of ecosystem structure affects ecosystem services (Balvanera et al. 2006) and resilience, the level of perturbation that an ecosystem can withstand without shifting to an alternative state that potentially provides fewer benefits to humans (Groffman et al. 2006). Canada has political responsibility for a large proportion of Earth's boreal forests, Arctic tundra, wetlands, and temperate and Arctic oceans, so better understanding of the structure and function of these ecosystems may inform Canadian decision making.

1. To what extent can ecological function and the supply of ecosystem services be predicted on the basis of ecosystem composition and structure?

2. How does managing ecosystems for particular ecological functions or services affect other elements of biodiversity?

3. How can transition points or thresholds among ecosystem states best be identified and predicted?

### Land Cover and Habitats

Current approaches to ecosystem management seek to link land-cover types, species’ habitats, and ecosystem stability and services in part to understand how natural and human-induced changes in land cover might affect ecological, social, and economic well-being (e.g., Ingraham & Foster 2008).

4. How are populations of animals and plants affected by different configurations of land cover?

5. At what spatial and temporal scales are management or policy interventions most likely to achieve various objectives for conservation of land cover?

6. What ecological principles and information standards employed in the identification of critical habitat under the Species at Risk Act are most likely to contribute to species recovery?

7. To what extent can habitat restoration or rehabilitation compensate for loss of quantity or quality of existing species’ habitat?

### Populations and Species

Many ecosystem functions and services depend on the maintenance of particular populations or species (e.g., Kremen et al. 2002; Lerdau & Slobodkin 2002). Populations and species are a focus of a variety of provincial and territorial legislation, such as the federal Species at Risk Act and fisheries management provisions under the Fisheries Act. Substantial investments are made by Canadian governments in managing species at risk, but Scott et al. (2010) note that meeting resource requirements for species that cannot recover without continuous management intervention could overwhelm organizations charged with protecting those species. Understanding the biology and dynamics of commercially targeted and nontargeted populations and species greatly increases the probability of effective species-oriented management.

8. What investments and management interventions will be required to maintain or increase the abundance of harvested populations when harvesting is one of multiple stressors acting on those populations?

9. What are the cumulative demographic and genetic effects of harvest on target and nontarget populations and species?

10. How do transboundary migrations of terrestrial and aquatic animals affect efforts to manage populations of those species?

### Resource-Based Industries

Natural-resource industries account for a substantial portion of Canada's economic activity (e.g., agriculture, food, and forestry industries account for over 10% of gross domestic product). Where past policies have failed to effectively manage biodiversity there have sometimes been enormous economic and social impacts. For example, collapse of the Newfoundland cod fishery led, in 1992, to the largest single layoff of workers in Canadian history (Rose 2007). Policy decisions related to the scope and operation of resource-based industries require broad analyses of the positive and negative impacts of interventions on biodiversity, the economy, and society.

11. What are the potential effects of large-scale energy exploration, development, and extraction projects on ecosystems and species?

12. How does management of terrestrial and aquatic food-production systems affect species, ecological functions and services, and economic benefits?

13. What are the magnitude and extent of effects of terrestrial resource-development activities on aquatic ecosystems?

14. How do extractive and nonextractive industrial activities affect ecological functions and biodiversity in aquatic ecosystems?

### Parks and Protected Areas

Canada has an extensive system of terrestrial parks and other protected areas (hereafter, parks) and a growing system of freshwater and marine parks for which federal, provincial, territorial, and regional governments all have responsibilities. Parks are managed to meet different objectives and mandates to protect species and ecosystems, and provide recreational opportunities that sometimes conflict. There are gaps in knowledge of how networks of parks can and should be designed, connected, and managed to meet ecological, social, cultural, and economic objectives.

15. To what extent can species be maintained over the long term within existing parks and protected areas in the absence of proactive, external management?

16. What are the ecological consequences of existing human activities in parks and their implications for future management?

17. What are the impacts of alternative configurations of, and management strategies for, aquatic reserves on ecological function, species, and social well-being?

18. What are the effects of different configurations of, and management strategies for, protected areas on biological diversity across landscapes and beyond the boundaries of terrestrial protected areas?

### Environmental Change

Canada is already affected by climate change, especially in northern regions (Prowse & Furgal 2009). Understanding the drivers and effects of rapid and extensive environmental change can help identify economic and social vulnerabilities (Ford & Pearce 2010) and inform the design of mitigation and adaptation policies at community to societal levels (Folke et al. 2005; Ford et al. 2010). Such understanding may also inform development of strategies and technologies that reduce the undesirable effects of environmental change globally (Government of Canada 2007).

19. How is climate change likely to affect the abundance, rates of growth, and distribution of and interactions among populations?

20. How can undesirable ecological, social, and economic effects of future changes in freshwater availability be reduced or mitigated?

21. How will northern coastal ecosystems respond to changes in climate and industrial activity if reduction in ice cover increases human access to those ecosystems?

22. What policies and management strategies most effectively mitigate the undesirable effects of non-native invasive species, potential disease vectors, and emerging pathogens?

23. What are the effects of different policies and management strategies on the capacities of humans and other species to adapt to environmental change?

### Environmental Values

Environmental values are influenced by demographic, cultural, experiential, and educational variables (Stern 2000). They can shape risk perceptions, behavior, and political support for conservation initiatives. Alternative approaches to addressing the root causes of human-induced biodiversity loss can vary greatly in effectiveness, costs, and benefits. Understanding demographic and cultural change and the range of alternative responses (e.g., education, communications, awareness building) available for different population segments may inform the design and successful implementation of stewardship and other conservation initiatives.

24. How does the integration of conservation science into public education influence environmental values, ecological literacy, and behaviors that affect the environment?

25. How does urbanization and immigration affect environmental values, behavior, and conservation outcomes?

26. How do individual and societal values about collective responsibility for common- pool resources affect private conservation initiatives and stewardship efforts?

### Economic Benefits and Costs

International agencies, governments, and researchers have highlighted the economic value of biodiversity. Accounting for the full costs and benefits of ecological change is central for credible economic analyses (TBS 2007). The way in which government departments and agencies share management responsibilities across a range of ecosystems and jurisdictions directly affects the cost of administration and management.

27. What are the true direct and indirect contributions of ecosystems to economic well-being and how are these benefits likely to change in the future?

28. What are the comparative ecological, economic, and social impacts of economic instruments versus input or output controls for ecosystem management?

29. What are the benefits and costs of horizontally and vertically integrating policies and regulations within and across different policy domains such as environment, health, and trade?

### Individual and Community Well-Being

Policymakers are concerned with aspects of individual, community, and societal well-being beyond economics. Environmental change affects human health (e.g., Myers & Patz 2009), communities and culture (e.g., Ford & Pearce 2010), and intangible factors such as individual, ethnic, and national identity (NRTEE 2003). In Canada particular consideration is ascribed by policymakers to individual and community well-being of Aboriginal peoples given Canadian legal obligations, Aboriginal peoples’ extensive reliance on biodiversity for their livelihood and community sustainability (including the maintenance of traditional ways of life), and the strong linkages between ecosystems and human health in rural communities.

30. How do terrestrial and aquatic conservation policies directly or indirectly affect human health?

31. What are the effects of conservation initiatives on the health, livelihoods, traditional cultures, and identities of Aboriginal peoples?

32. What are the effects of different strategies for building community capacity on levels of citizen engagement in environmental stewardship, restoration, and conservation?

### Adaptive Management

Canadian governments are committed to working together with citizens using “an ecosystem and adaptive management approach to achieve shared outcomes” (Environment Canada 2006). Ecosystem management seeks to increase the probability that management will achieve ecological, social, and economic objectives by incorporating evidence generated by directed policy experiments (Rudd 2004; Folke et al. 2005). Understanding and clarifying what constitutes evidence and when and how various forms of evidence are used in the adaptive management process are crucial for informing ecosystem-management decisions. In some instances, “sufficiently sound scientific information” for decision making can be derived from traditional knowledge (Government of Canada 2001).

33. How can local and traditional knowledge be most effectively communicated and synthesized with scientific knowledge to inform conservation science and management?

34. What forms of scientific evidence, risk assessment, and knowledge transfer most effectively and increase the probability of achieving ecosystem-management objectives?

35. What monitoring methods can effectively and efficiently assess long-term, incremental, and cumulative environmental changes?

36. What are appropriate indicators to evaluate the effects of public, private, and nongovernmental-sector policies, programs, and initiatives on biodiversity?

### Policy and Governance

Canada is a parliamentary democracy with a complex federal structure and diverse citizens who expect to participate in collective decisions. This situation complicates the formation and implementation of national evidence-based and results-oriented policy. It is difficult to develop consistent policies across jurisdictions and to determine the most-appropriate level of governance to address a given environmental problem. It is also difficult to balance the legitimacy of governance (a function of structure and inclusive processes) with explicit action to conserve biodiversity as required by laws, international commitments, and moral values.

37. What factors influence the likelihood of compliance with environmental legislation and regulations?

38. How do policy, legal, or institutional arrangements shape the effectiveness of integrated management for terrestrial watersheds and adjacent coastal environments?

39. How do the actions of Aboriginal governments and organizations influence conservation strategies and the assignment of responsibilities for environmental governance in Canada?

40. How can environmental laws, policies, and regulations be integrated and harmonized across multiple jurisdictions and sectors to achieve conservation objectives?

## Discussion

Answers to questions about conservation policy can be informed by scientific evidence about the ecological, social, and economic effects of governance interventions. The Government of Canada (2000) identified six general principles of how scientific advice should be sought and applied to enhance the ability of government decision makers to make informed decisions. They include communicating among decision makers, policy advisors, and scientists for identification of issues; using a diversity of scientific schools of thought and opinion (including experiential or traditional knowledge) to enhance inclusiveness; ensuring rigorous review so that sound science and science advice is available to decision makers; assessing, communicating, and managing uncertainty and risk; maintaining transparency and openness to demonstrate how science was taken into account in decision making or policy formulation; and reviewing key decisions to determine whether advances in knowledge affect scientific advice and warrant policy or managerial adaptation. Our collaborative exercise illustrates how both the process and outputs of research prioritization can be used to support these widely applicable principles of good governance.

Some of the themes identified in this Canadian exercise coincide closely with those identified in the United States (Fleishman et al., unpublished data). Relative to the United States, however, there was less emphasis on environmental stressors (27% vs. 55% of the 40 questions) and more emphasis on governance and management issues (67% vs. 32% of the 40 questions). In Canada there was strong emphasis on the effect of aboriginal governance on federal and provincial conservation policy and, conversely, on the effect of governmental conservation policy on individual and community well-being for Canada's Aboriginal population.

We suspect that the contrasting priorities within North America arise from cultural and political differences (i.e., consensus, policy harmonization, and the nature of federalism may be more important in the Canadian context than the “checks and balances” of the American system). In other fields of study, political context has been identified as an important factor influencing the effect of research (Shulha & Cousins 1997; Rigby 2005), so it would not be surprising if research priorities differed among decision makers working within different political regimes. It is also possible that there is less need for information about environmental stressors in Canada, a country with low population density and less human activity, leaving room for more governance-oriented questions. In future national-level exercises, it may be possible to discern research priorities that are common across most nations and those that vary depending on internal levels of environmental stress and on the type of governance regime.

The questions we identified provide potential links between evidence from the conservation sciences and the formulation of environmental policies for conservation and resource management. Integrating knowledge from across the natural and social sciences is necessary to effectively address societal tradeoffs between use and conservation of biodiversity. The collaborative process of communication and engagement between scientists and decision makers can change the ways that decision makers think about evidence, enhance levels of mutual trust and credibility, and potentially help advance the conservation policy discourse in Canada and internationally.
