# Supplementary material for: Generation of Priority Research Questions to Inform Conservation Policy and Management at a National Level
Source: Conserv Biol. 2011 Jun;25(3):476–84. doi: 10.1111/j.1523-1739.2010.01625.x (PMC3108069; doi:10.1111/j.1523-1739.2010.01625.x)
Supplement: Supplementary file 1 [file cobi0025-0476-SD1.doc]

**Notes:**

Contributors of candidate questions could opt to be identified (in parantheses). Several manuscript co-authors as listed as question contributors. Questions were solicited before workshop participation was finalized. Contributor names were not disclosed to workshop participants.

French candidate submissions were translated to English for this summary.

Some minor editorial changes were made to several candidate submissions. These involved either minor grammar corrections or rephrasing a statement into a question.

**Bold** font indicates that the question was considered in depth by breakout groups at the Ottawa workshop. Some of the questions not specifically considered were deemed (by the core team prior to the workshop) too broad or too narrow, not specific to Canada, or redundant. All 396 questions submitted for the exercise were available at the Ottawa workshop for consideration by the participants and could be brought forward at any time.

***Bold and Italic*** font indicates that the question was selected as one of 56 Top 40 candidates at the Ottawa workshop. The total shown here is less than 56 because breakout groups synthesized some new questions that drew on ideas and themes within a topic area.

1. **In freshwater recreational systems, can protected areas be designed that will allow multiple uses in a sustainable manner? (Hedrik Wachelka)**
2. **How are species, and in particular species at risk, distributions being impacted by the rate of climate change and the fragmentation and habitat loss of regions to which they are colonizing as a result of climate change? (Laura Coristine)**
3. **What are the effects of human-induced landscape change (i.e. habitat loss, fragmentation) on biodiversity?**
4. **How do diseases of domestic game animals, fish, and livestock affect wild populations?**
5. **How can species diversity be maintained in the face of hydropower development? (Bryan Allemang)**
6. What is Canada’s role in conserving and protecting ocean and aquatic environments and what actions are required to achieve it?
7. ***How can we better integrate (in terms of research, policy, administration, evaluation and innovation) both horizontally (across policy domains) and vertically (across levels of governance and jurisdictions) to link conservation and environmental action with other policy areas?***
8. **Using an international comparative approach, which national conservation strategies have been most successful, and what aspects of those strategies have made the most effective contribution to that success? (Martin Willison)**
9. How can scientific knowledge be abstracted for both long term planning and everyday remedial action in integrated coastal management? (Daniel MacInnes)
10. Would standing interpretative councils representative of users, scientists and managers work at the regional coastal management and, if so, would national deployment require further legislation for establishing arms-length independence?
11. **How do various forest management activities affect structure, composition and function of forests?**
12. **How would NGOs, Government agencies, and academia benefit from utilizing information such as satellite imagery to effectively monitor and detect changes in ecosystems nationally and globally to make timely and accurate policy decisions? (Hui Wei)**
13. How many ships are ice-strengthened or have ice-breaking capabilities and what risks does increased navigation pose in the Arctic environment? (Laura Tucker)
14. ***How can the potential off-shore aquaculture activities be integrated with today's commercial fishing activities to assure that the wild fisheries remain sustainable and the ecosystems healthy? (Leslie-Anne Davidson)***
15. **What are the socioeconomic benefits of MPAs in northern waters?**
16. **What level of (a) fishing capacity reductions and (b) area protection per habitat type are required to move Canada's use of ocean resources to a sustainable level within an ecosystem based management approach under the growing influence of climate change? (Dirk Zeller)**
17. What are the true costs and benefits (including social, cultural, economic and environmental; direct, indirect and cumulative ) of mining, selling, and burning oil derived from the Alberta tar sands? (Irene Novaczek)
18. **What is the value of biodiversity, ecosystem services and natural capital in Canada?**
19. ***Do mechanisms or schemes to manage ecosystems for a particular ecological function or service tend to have negative or positive effects on biodiversity and, if so, which policies, management conditions and/or actions tend to create negative or positive effects? (Thomas Hammond)***
20. How can federal laws and policies pertaining to marine conservation, such as the Species at Risk Act or the Federal Marine Protected Areas Strategy, successfully achieve their objectives despite the socio-economic consequences associated with their implementation? (Chris Burbidge)
21. **How do we best quantify the direct and indirect benefits to Canadians of intact (e.g wilderness) and semi-intact (e.g. parks in degraded matrix) ecosystems such that these benefits can be directly compared to the direct and indirect costs of the creation, management and preservation of these areas? (Arne Mooers)**
22. **What are the costs or implications of the loss or alteration of our natural ecological systems for short- and long-term human health? (Ruth Waldick)**
23. ***What are the key factors that promote responsible stewardship on private lands?***
24. **What data systems will need to be accessed and integrated in order to address the impacts of climate change, environmental impact assessment and other related multidisciplinary environmental priorities?**
25. **What will it take for all stakeholders at all levels of jurisdiction to agree on rooting resource management policy and practice, for every watershed, firmly within ecosystem based management and traditional knowledge?**
26. ***How do we reconcile the rights, values and land uses of the Aboriginal peoples of Canada with those of the Canadian state, in particular the provinces, which have constitutional responsibility for natural resource management? (Peggy Smith)***
27. **How do we identify critical habitats under SARA? Resource selection functions can identify areas used by animals, and similar models can identify distributions of plants. But ultimately we need to understand fitness associated with various habitats. How do we do this efficiently? (Mark Boyce)**
28. What is the maximum sustainable allowable cut of trees provincially and federally to ensure healthy forest ecosystems? (Brennan Anstey)
29. What are the direct relationships of biological diversity with the lives of Canadians?
30. ***How can agricultural areas be managed to maintain species and ecosystem services while still being economically viable? (Nora Szabo)***
31. **How can the amount and direct impact of plastics pollution in the ocean be effectively monitored? (Aidan Wind)**
32. **What strategies can best be used to reduce impervious surface and associated storm water run-off in coastal areas? (Jesse Sayles)**
33. **How can private land conservation efforts best be integrated with public land conservation programs?**
34. ***What impacts will climate change have on our Canadian network of protected areas and how can we proactively adjust for these changes?***
35. **What is the relationship between conservation policies that relate to a system model (Parks Canada) versus ones are location-specific (e.g., DFO, biosphere reserves)?**
36. **How can local ecological knowledge (LEK) better inform understandings and sustainable use of ecosystems? (Anthony Davis)**
37. What are the most reliable approaches to understanding LEK? (Anthony Davis)
38. **What are the best approaches to enabling sustainable conservation through empowering local stewardship?**
39. **What is the importance – in terms of attitudes, beliefs, values and behaviour – of natural environments to Canadians? (Alistair Bath)**
40. **Can we determine and manage human population and impact (i.e., ecological footprint) targets to ensure we maintain sustainable landscapes and ecosystems? (James Duncan)**
41. **As habitat loss and degradation is a key threat to species survival and recovery, how do we apply a provincial land use plan to deal with multiple species biological and ecological needs as well as economic and social needs? What data is required and can it address critical habitat concerns?**
42. What is the economic value of critical ecosystem services in Canada?
43. **How can we make conservation strategies economically profitable for those affected, such as resource industries and farmers?**
44. How can we effectively bring tourism and conservation together?
45. How can protected area networks be developed and designed to account for and adapt to changing species numbers and distribution that will result from climate change?
46. **Are marine protected areas a sufficient tool to restore and maintain commercial fisheries?**
47. **Is boreal forest open to invasion by insects?**
48. **Which social, economic, and governance characteristics best promote urban and rural community-based wildlife conservation? (Glen Hvenegaard)**
49. ***What are the economic and social values of ecosystem services and how can these be incorporated into management priorities? (John Reynolds)***
50. **How do we reconcile Canadians’ ethics, values, and opinions regarding species preservation goals with constraints imposed by the economic costs to satisfy those goals?**
51. **What is the appropriate governance structure that enables placed-based sustainable natural resource programs and policies to be successfully implemented?**
52. How do ecosystems interact and how can be better manage/protect so they can thrive and provide us with ecosystem services? (Vivek Voora)
53. What affect will the projected growth of human populations have on the natural environment in Canada?
54. What are the effects on governmental resources policies (fisheries, oceans, forests, mining) on the human dimensions (economics, social, political) of conservation?
55. **How much, and a what rate (i.e., gradual or in bursts), will increased melting of the tundra contribute to increased levels of atmospheric CO2?**
56. **What science-based strategies from government and educational institutions can incorporate conservation (which includes the management of species) into the school curriculum in order for the teachers and students to better understand ecosystems and the outdoor environment?**
57. **How can science contribute to reconnecting humans to the ecosystems that we so depend on for our survival? Conversely, how have humans become so disconnected from the ecosystems that we so depend on for our survival? (Colin Soskolne)**
58. What are the factors that explain the distribution and abundance of species and the interactions between species and their environments?
59. Why do Canadians claim that they value nature (or at least have the north, the Rockies, etc., as part of their national identity) and yet they systematically replace it with artificial systems at every opportunity?
60. **If large marine predators (whales) rebound to historic or near historic levels, what impact will this have on existing commercially fished stocks? Alternatively, how much do commercial fish catches have to be reduced to provide enough forage fish biomass to promote recovery of large baleen whales? (Sean MacConnachie)**
61. **How do American and Canadian environmental laws affect fringe species (i.e. species who are common in the US or Canada but whose distribution across a border makes them rare in the other nation)?**
62. ***What locations, and proportion of area, of marine reserves in Canada's three oceans would adequately protect marine biodiversity and allow sustainable marine capture fisheries?***
63. Is the exotic pet industry a significant source of disease for our natural ecosystems? (Ian Kanda)
64. What is the biotic response to ecosystem restoration efforts?
65. ***What types of species are most vulnerable to the combination of climate change, habitat loss, and habitat isolation? i.e., what combination of life history traits, phylogenetic history, and geographic location can predict species vulnerability? (Jay Fitzsimmons)***
66. How has human influence impacted biodiversity in sensitive ecosystems?
67. **What factors or barriers limit the use of scientific information in environmental legislation and policy implementation?**
68. **How can provincial and federal environmental legislation be harmonized?**
69. **How does one assure that local endemic biodiversity is protected as well as high mobile and migratory species?**
70. **How do factors such as habitat type, habitat quantity, and landscape configuration impact the migratory movements of birds across the North America at different scales?**
71. What would constitute appropriate recovery targets for abundance/density and distribution for depleted populations and species? (Jeffrey Hutchings)
72. **What are the cumulative impacts of environmental change (development, climate change etc) on Pacific salmon driven ecosystems and what mitigation procedures could be most effective?**
73. How better to integrate the socioeconomic and environmental dimensions into the territorial planning activities and what are the roles of governments and other groups?
74. How can non-governmental organizations effectively participate in environmental management processes?
75. What are the zones of critical coastal vulnerability, which measures would reduce that vulnerability and to assure ecosystem sustainability, and which environmental and socioeconomic indicators would allow to assess the effectiveness of those measures?
76. **Is the re-location of plant populations feasible and, if so, how can the viability of transplants be assessed?**
77. **What are the minimum habitat and resources necessary for humans to survive and prosper across Canada and how can we re-think conservation policies to limit our expansion beyond that?**
78. **What are the expected decadal scale impacts of anthropogenic climate change on terrestrial, marine, and freshwater ecosystems? (Ian L. Jones)**
79. What is the primary cause of increased temperatures worldwide? (Melissa Sawchuk)
80. **Which populations at the northern range peripheries are genetically or ecologically distinct relative to the rest of the species' range and which are likely to foster range expansions under climate change?**
81. How resilient are ecosystems and how does this information get used to develop adaptive conservation strategies? (Nigel Roulet)
82. ***What current and expected changes in freshwater lake ecosystem are due to the combined effects of expanding exotic species (e.g., zebra mussels) and climate change?***
83. **What are stakeholders' primary objectives for the natural resources they use and how do these differ from the goals and objectives of the general public? (Andres Cisneros-Montemayor)**
84. **What level of land preservation and financial investments are socially and economically acceptable in order to preserve biodiversity in Canada?**
85. ***What policy, legal or institutional changes need to be made to link land/watershed management to coastal/ocean management?***
86. **What are the institutional arrangements for collaborative management on aboriginal land that lead to effective conservation?**
87. **Which multinational governance, cross‐sector cooperation arrangements, and finance mechanisms will make highly migratory marine mammal and turtle species management more effective?**
88. **How can mitigation and adaptation projects in Canada be improved so that First Nations benefit more from the economic opportunities of transitioning to a sustainable economy, thereby improving social conditions in First Nations communities while contributing to Canada's GDP? (Tracy Coates)**
89. **How can the protection of at-risk habitats be pursued in an equitable way across different jurisdictions, such as federal lands, including Indian reserves; provincial Crown lands, including traditional territories and resource tenures; municipal lands; and, private lands?**
90. What are the habitat roles of small temporary wetlands in maintaining amphibian populations? (Godwin Okonkwo)
91. How have past monitoring strategies contributed to overall ecosystem health and management strategies to preserve overall biodiversity?
92. **To what degree does vessel noise interfere with the prey detection of echo-locating whales and dolphins and does this disturbance have a population level effect? (Doug Sandilands)**
93. **How can all the natural communities in Canada be arranged in a hierarchical classification system than facilitates conservation decision-making? (Robert Alvo)**
94. **How can we stop aquatic invasive species from entering the Great Lakes freshwater ecosystem, and minimize the impacts of the current mass of alien invasive species in this system? (Ian L. Jones)**
95. What are the boundaries we need to define temporal scales of change given the multiple stressors and inherent non-linearities in ecosystem structure and function? (Nigel Roulet)
96. **What are the relative effects of vessel noise compared with vessel presence towards disturbing cetaceans? For example, are killer whales affected by the presence of kayaks (or sailing vessels) to a greater or lesser degree than by motorized vessels under power? (Doug Sandilands)**
97. Is capitalism fundamentally at odds with environmental conservation, and if so, what are we to do about it? (Ian L. Jones)
98. What is the relative importance of vessel disturbance compared with declines in prey abundance and high toxin loads in the survival of cetaceans and is there a negative synergistic effect between vessel disturbance and low prey abundance and high toxin loads? (Doug Sandilands)
99. What is an accurate ranking and/or valuation of human impacts on the environment? (i.e. not just looking at jobs created, and value of resources generated by activities, but balancing it with ecosystem disruption, wildlife losses, and habitat irrevocably destroyed). (James MacKay)
100. How can we reconcile environmental education and values to actions by industry, governments and other organizations? (i.e., are there incentives that can be linked to ecosystem conservation and could effectively move agendas such as the Biodiversity Convention or Kyoto Protocol?)
101. How can we incorporate the sacred or spiritual dimension and our love/connection/relationship to 'environment' and 'resources' into policy and decision-making that meets the criteria of eco-justice (solution for ecology and poverty has to be one and the same)? (Nigel Haggan)
102. **What residual stream flows that vary according to season and location sustain healthy ecosystems and fisheries productivity? (Ron Ptolemy)**
103. ***How much water must be maintained in streams and rivers subject to human withdrawals to prevent ecosystem degradation?***
104. What can be done to protect stocks of Pacific salmonids against future climatic warming?
105. ***How can we best incorporate uncertainty in current estimates of abundance or biomass of living resources (e.g., fish stocks, caribou herds, etc.) in ecological models and harvest quotas?***
106. **What factors affect the productivity of Pacific salmon in the marine ecosystem and by what degree?**
107. **What is causing the major decline in marine survival of salmon and can it be compensated for by freshwater interventions? (David Welch)**
108. **How does the sub-population structure of species (e.g. Pacific salmons) contribute to their resilience and adaptability in response to environmental change? (Mike Morrell)**
109. **What are the limits to human degradation of water quality in freshwater and marine systems that result in ecosystem shifts?**
110. ***What management interventions will be required to restore depleted and endangered marine fish stocks off Canada's east coast?***
111. How do we define and quantify environmental diversity and its linkages with biological diversity?
112. How can be limit urban sprawl to protect species?
113. Why is biodiversity important?
114. What activities can prevent or mitigate harmful alterations of anthropogenic activities on productive habitats of fish and wildlife? (Steven Kerr)
115. **What adaptation strategies are likely to have the greatest ability to mitigate / reduce the effects of climate change on marine coastal ecosystems?**
116. **Why are environmental data collected by various levels of government not centrally located and available to allow environmental management decisions to be made in a more ecosystem-based, rather than site specific and ad hoc, manner? (Edward Malindzak)**
117. **What are the effects of hydroelectric power generation on aquatic resources? (peaking and ramping as well as additional work on the establishment of "ecological" flows).**
118. **How will climate change affect aquatic species and their ecosystems in the biomes across Canada and the Great Lakes Basin?**
119. What fish species use the tributaries of the Great Lakes? (much of the information collected on these assemblages is at least 30 years old)
120. What are we willing to give up today, to protect our environment for the next generations?
121. What will drive people to make the tough decisions and re-evaluate the concept of sustainable development in perpetuity?
122. How do the cumulative disturbances to headwater systems impact on the ecological integrity of aquatic ecosystems? (Les Stanfield)
123. How can government regulate corporations such that they can have a smaller ecological footprint while still remaining competitive?
124. What is an appropriate approach to establishing minimum flows (baseflow) at hydroelectric facilities in a manner that optimizes the balance between efficient megawatt production and retention of viable functioning aquatic ecosystems in the downstream environment? (Steve McGovern)
125. How can we manage the cumulative effects of development on the environment?
126. What criteria must be considered to make decisions in establishing conservation policies? (Melanie Rousseau)
127. **What are the ramifications of the Canadian approach to governance and the indirect impact on the value of ecological assets compared to other models of governance/democracy?**
128. **What is the link between the quality and quantity of fish habitat and fish production?**
129. How are our economy and ecosystems in Canada practically inter-related and interdependent?
130. **How much habitat space is needed to protect all essential ecological systems in Canada's biomes? (Ken Minns)**
131. **In freshwaters, what portion of Canada's lake resources in the Boreal and Taiga biomes must be set aside to ensure the persistence of the range and abundance of the main freshwater fishery species?**
132. How can Canada incorporate ecosystem based management in all decision making?
133. **How can we develop, validate, and refine predictive models of the relationships between flow and biological attributes of streams (e.g., fish and invertebrate production, diversity, and community structure)?**
134. **What role do small, ephemeral, unprotected wetlands play in the environment in terms of contributing to biodiversity, providing habitat for rare and endangered species, hydrological processes, and genetic fitness/population connectivity? (Elke Wind)**
135. **How can cumulative effects on species and ecosystems be accounted for in individual permitting decisions?**
136. ***How do we assess and manage cumulative impacts of both human activities and natural disturbances in an ecosystem context? (Keith Clarke)***
137. Can the biota adjust to the climate change without considerable loss in biodiversity?
138. Assuming that spill-over / boundary effects of marine reserves enhance fish catch, what is the optimal percent area in no-take vs. harvested zones to maximize catch per unit of effort and what factors (e.g., mobility of different harvested species at different life stages) influence this determination? (Ole Hendrickson)
139. What is the carrying capacity for human development on watersheds? (Sackville Rivers Association)
140. How to measure the vigour with which energy flows through the aquatic ecosystem (trophic efficiency) such that aquatic habitat / ecosystem values can be quantified? (Gary Swanson)
141. ***What are the levels of monitoring and enforcement necessary to secure functional conformity with regulations related to a variety of types of reserves, and what are realistic estimates of the costs and benefits of such monitoring at the local, regional, national, and global scales?***
142. ***Given the current scientific literature is lukewarm on habitat creation and yet many species are opting for anthropogenic habitats, for which taxa and in which ecosystem contexts can habitat be successfully re-created as offset mitigation for habitat destruction?***
143. **What happens to the Atlantic salmon at sea? (Sackville Rivers Association)**
144. **How does acid rain impact river water quality and biodiversity in the Nova Scotia southern uplands and are there mitigation measures (i.e., liming) that can counteract these impacts? (Sackville Rivers Association)**
145. What are the best construction practices for working on drumlins (fine clays)? (Sackville Rivers Association)
146. Can we better gauge river flows using satellites? (Sackville Rivers Association)
147. Why are American eel populations in Canada declining? (Sackville Rivers Association)
148. **What is the economic value of ecosystem service protection in Canada and how can we internalize externalities associated with the environment?**
149. **What is the best strategy to assure connectivity between protected areas?**
150. **What are the likely future changes in habitat for Canadian wildlife (e.g., movement northward; reduction of habitat; decline in quality)? (Nancy Kingsbury)**
151. **How can we measure the benefit, compared to the costs, to Canadians of a functioning ecosystem?**
152. **How effective are MPA's as a managment tool (socially, economically, and culturally) in advancing Integrated Fisheries Management in Canada and what are the various assessment tools being used? (Virginia Boudreau)**
153. Can the "50/500" rule (and perhaps other general "rules of thumb") be revisited and revised to better inform recovery planning for Canadian species at risk in the context of setting population and distribution objectives and critical habitat identification under SARA?
154. **How much of the knowledge and research ability of environmental consultants be financially uncoupled from the vested interests of private industry in order to realize its full potential for society at large? (Kathy Mai)**
155. ***What is the current condition of freshwater resources, what is the trend, and what are the best biological indicators?***
156. ***What thresholds of wetland cover, immature forest and impervious surface are critical to maintaining watershed health?***
157. **To what extent has professional reliance and a results-based legislative framework in British Columbia improved management and conservation of species and ecosystems at risk?**
158. **What are the core monitoring requirements necessary to: 1) assess the condition (ecological integrity) of coastal and marine ecosystems and how conditions change over time; 2) evaluate the effectiveness of MPA networks; and 3) evaluate specific MPA/Marine Reserve (no-take) networks design and management? (Ann McCrone)**
159. **How can we implement multi-scale, resilient governance systems to address multiple and often conflicting priorities while safeguarding the integrity of natural systems? (Karen Morrison)**
160. What role do headwater drainage systems (0 order; intermittent; ephemeral) play in the provision of fish habitat (direct and contributing to), water quality and water quality management and how effective are mitigation measures in replicating these functions? (Deborah Martin-Downs)
161. How do species fit in ecosystems and what role they play?
162. ***What are the most effective ways to educate the public and engage them in stewardship of species and ecosystems?***
163. **What are the differential impacts of species versus system management?**
164. What is the role and impact of buffers around protected areas?
165. **How can cumulative effects of marine uses and human activities of varying intensities be measured using appropriate criteria and indicators? (Nicole Schaefer)**
166. What is the appropriate role for early involvement of local citizens in the siting of wind turbines? (Carl Littleton)
167. ***How do we build capacity in communities for environmental protection, conservation and restoration?***
168. How do we conserve the biodiversity of the Canadian boreal region in light of the tremendous anthropogenic threats in the region? (Shawn Leroux)
169. **What are the ranges and life history patterns of populations at risk (including data deficient populations), and how can Canadians monitor and protect these systems?**
170. ***How do we identify critical habitat likely to impact SARA species and establish thresholds for activities that are likely to destroy that critical habitat?***
171. **What wetland types have the highest historic losses and how do they differ regionally?**
172. **How do managers determine the optimal proportion of habitat to protect? (Ann McCrone)**
173. What is the assimilative capacity of streams with regards to urban stormwater discharge and how can this knowledge guide the development of policy and mitigation measures to protect aquatic functions, particularly for aquatic species at risk? (Deborah Martin-Downs)
174. For both human and animal species, what are the health effects of low frequency sound? (Carl Littleton)
175. **What are the rates of carbon sequestration for different wetland types in Nova Scotia?**
176. What are the best biological indicators of wetland ecosystem condition for different types of wetlands in Nova Scotia?
177. **How can the role of wetlands in moderating climate change best be characterized?**
178. **How can scientists and policy-makers effectively draw on dynamic local and Indigenous knowledge in the context of cooperative, adaptive management and governance in order to strive for perpetual ecosystem health in a changing climate and uncertain future?**
179. **How can protected area networks be designed to increase carbon storage benefits and mitigate climate impacts, with these benefits as incentives to support conservation actions?**
180. **What levels of functional tolerance can be sustained through existing land use practices and what are the levels of ecological resilience to these changes? (Mark F. Elderkin)**
181. What is the mandate of conservation organizations in an era of global climate change? (Daniel Scott)
182. **None! What is needed is dramatically improved inventories for species and ecological communities of conservation concern (including those at risk). Inventories for communities of conservation concern require that those communities be defined in detail (far from complete in Canada) and then ranked. (Dr. R.A. Lautenschlager)**
183. **How can we understand the impact assessment of arts in environmental learning to contribute to a social shift of environmental responsibility with respect to resource management and ecosystem services? (Tascha Stubbs)**
184. ***Where are the most strategic places for future conservation that would 1)provide linkages between existing protected areas, 2) buffer the existing system and 3) add to our carbon management plan? (Tory Stevens)***
185. **How are ocean ecosystems structured, and how do perturbations in one component impact the valued species coastal communities depend upon? (Fred Whoriskey)**
186. **What is the extent of responsibility, legally, morally and ethically, that land owners do hold, should hold and/or can exercise (or should be made to exercise) regarding the protection/conservation/stewardship of species, ecosystems and ecological processes on their respective lands? (Dale Smith)**
187. **What are core Canadian values with respect to the environment (and our place in it), and to what degree are those values reflected in personal and collective behaviour (particularly in government policy as an expression of public will in a democratic society)?**
188. How will future changes in the distribution of human impacts on ecosystems (via land use) affect the viability of valued species and habitats?
189. How does music facilitate sustainability education, intensify environmental concern and action among students and, through intergenerational transfer, transfer this motivation to their parents? (Holly Arntzen)
190. **What is the environmental impact and "carbon footprint" of OHV's and ATV's in wilderness areas? (Jim Barlow)**
191. **How do we integrate the concept of ecological integrity into Canadian policy (federal, provincial, municipal) and how is this concept associated with the well-being (and health) of people? (Daniel Rainham)**
192. What are the effects of climate change on inland cold water fisheries?
193. **What programs are being developed to closely study/monitor sea-level rise, its impact on low-lying areas and related species, and what programs will be put in place to mitigate/remediate effects of rising sea-levels?**
194. **What is the best method of ensuring that the public's true desires and interests are reflected and incorporated in policy decisions around endangered species habitat protection?**
195. **How do we rigorously assess the weight of scientific evidence associated with the set of causal hypotheses on which candidate policies, management interventions, statutes etc are (ostensibly) based? (Scott Findlay)**
196. **How important is it to have subsurface rights within a protected area withdrawn from industrial development?**
197. **What are the best predictors of success for a protected area being able to conserve biodiversity in the long term? (Stephen Woodley)**
198. ***What social and ecological vulnerabilities are produced through a greater dependence on private sector funding for conservation initiatives?***
199. **What are the spatial and temporal scales at which we must study the interface between policy implementation and impacts of human activity on the environment (agriculture, forestry, urbanization)?**
200. What are the criteria for an effective MPA network? (Jason Simms)
201. **Why are aboriginal people in northern Canada restricted by new laws (because of climate change)from their inherent right to harvest food from natural resources? [people from northern Canada have very little input to the cause of global warming compared to the sprawling industrial cities].**
202. **How can we best incorporate the value (social, economic, cultural, spiritual) of ecosystems into environmental and economic policies?**
203. ***The role of deepwater coral, sponge and other benthic ecosystems in overall marine ecosystem function and how are fishing activities impacting that ecosystem function?***
204. **How do land-based activities (such as run-off, etc. from agriculture, mining, peat harvesting, sewage, forestry, etc.) affect marine ecosystems?**
205. **What are the main barriers to changing the behaviours of Members of Cabinet, elected members of the government of the day, senior members of their civil service, to support effective policies? (Wayne Barchard)**
206. How much and what type of natural areas conservation is needed to sustain biodiversity and ecological processes in the future?
207. **How do we make science-based conservation relevant to politicians and society?**
208. **Real action on climate change is essential for almost any set of conservation policy to be successful. With this in mind the elemental research question then has to investigate socio-politcal motivations that aid or inhibit social change toward a low-carbon society.**
209. How relevant and applicable are high product and process standards developed by major firms in industries such as chemicals and mining for the thousands of smaller firms in these industries? To the extent they are, how well do these smaller firms conform?
210. **How do species distributions (native and exotic) change at local and regional scales over time, and what is the best method to track these changes? (Steve Hill)**
211. What aspects of the human vectoring of invasive plant species can be effectively utilized to assist in the resilient migration of rarer plant species in the face of climate change?
212. With the advance of many invasive insects and plants in Ontario, what is the governments role in identifying the most destructive organisms?
213. What are the most ecologically sound practices in all sectors and what is the most efficient strategy for enabling the public to follow these best practices? (Anya Reid)
214. **How much connected, healthy habitat (terrestrial and aquatic systems) do we need to protect in order for species to continue to thrive/rebound? (Shannon Miedema)**
215. ***What are the contributions that natural systems make to the economy of our country? [real numbers and valuation of natural assets vs. direct economic use/extraction of those assets]***
216. **How can we increase personal connection to nature so people feel integrated, protective and have a sense of wanting to regenerate their local environment while maintaining the ecological integrity of natural places and habitat for species? (Laura Alward)**
217. **What are the true monetary costs and benefits of a) declaring a species at risk; and b) protecting large natural areas for wildlife habitat? Do those costs and benefits change over time and across space in Canada?**
218. What long-term environmental changes are being caused by human activities, how will this be reflected in the ecosystem and what can be done to mitigate these changes so as to minimize impact on valuable resources? (Clarence Bourque)
219. **What alternative governance models could be implemented in Canada to truly establish sustainable communities considering the holistic and inter-related reality of nature and the dependence of economies and societies on healthy ecosystems and fair use and allocation of the Earth's resources?**
220. To what extent is public opinion considered when making decisions regarding ecosystem policy?
221. **How can the public become more engaged in marine conservation science and planning efforts in Canada, and be given a sense of stewardship of the environment?**
222. **How can environmental costs be effectively factored into the overall cost of a project in such a way that it will be accepted and adopted by proponents, decision makers, and policy makers?**
223. How can we effectively manage our use of the oceans to prevent further species and ecosystem collapse? (Louise de Mestral Bezanson)
224. **What are the cumulative impacts of human stressors on Canadian marine ecosystems? (Michelle Greenlaw)**
225. **What areas could be inundated by storm surge / tsunamis / natural hazards and how can they be mapped?**
226. How can we define a performance management framework that incorporates the scientific (biological and climatic) advice with the social, economic and political factors and provides an estimate of the relative impact of the 4 factors that are used to make resource management decisions?
227. What combination of sustainable resource extraction and sustainable conservation actions provide a balance between socioeconomic and biodiversity criteria that is scientifically based and socially acceptable?
228. **How forest tenure system would encourage management for long-term forest sustainability? (Rob Rempel)**
229. **How can we determine historical, unexploited population structures (numbers at age, size, sex) of components of a given ecosystem, where direct and/or indirect influences of exploitation precede the onset of scientific monitoring (i.e., what did a balanced ecosystem look like)?**
230. **How can we create opportunities for the scientific, academic, development, and policy communities in Canada and developing countries to share results and experiences on sustainable development?**
231. **How do we develop food production systems that are capable of meeting our societal objectives of food production and coastal revenues while being environmentally benign and able to exist within natural ecosystems? (Shawn Robinson)**
232. How is the reduction of permafrost influencing water availability and chemical spreading in soil and in the atmosphere? (Michele Leone)
233. **What conservation efforts can be made to reduce the impact of wildlife link to emerging infectious diseases transmission? (Lola Lawrence)**
234. How can we accurately account for cumulative effects on marine ecosystems of the various human activities that occur in the near-shore coastal zone?
235. Is it truly a lack of certainty & sufficient information or is it something else?
236. **Which are drivers for eco-health services impacting on biodiversity, how does adaptation/mitigation (e.g. biofuel) to climate change impact on these, and how do these causal chains impact on emerging disease patterns of risks and vulnerabilities?**
237. **To what extent does post-release mortality influence population abundance and productivity of both commercially-fished and non-commercial fish species which are discarded? (Steven Campana)**
238. How do we develop a better understanding of nearshore ecosystems, which are the most vulnerable to human impact?
239. What affects the long-term stability of populations and ecological communities? (Kurtis Trzcinski)
240. How can exemplary educational programs and practices now carried out in schools and communities be financially supported and sustained?
241. ***How does ecosystem function and the quality/quantity of natural cover benefit human health and well being?***
242. **Can we develop new methods for determining the spatial extent and temporal persistence of organic pollution in the marine environment (e.g., applicable to aquaculture, pulp and paper and sewage wastes)? (David Wildish)**
243. **Do Canada's provincial and federal environmental protection regulations and enforcement efforts actually protect the environment?**
244. **Can we gather annual abundance and distribution data annually – for all "harvested" wildlife, fish and forest species and key/representative non-harvested species – that can provide the foundation for policies and management strategies?**
245. **What are the causal links between human-use activities and pressures that can be manifested as environmental effects in aquatic ecosystems? What is an appropriate burden of proof form a social perspective to provoke actions on the basis of a reasonable assumption of a cause-effect link?**
246. **Aside from obvious environmental challenges, what other challenges may the melting of the Arctic ice impose on Canada?**
247. **At multiple scales, what cumulative impacts are most appropriate as measures (indicators) of risk to species, ecosystems and ecosystem processes? (Mark F. Elderkin)**
248. **What land use planning tools currently exist or could be developed to provide direction to the conservation of species, ecosystems and/or ecological processes found on private lands in Canada? (Dale Smith)**
249. How do artists as messengers, and cultural events as platforms (i.e., community music concerts), achieve highly effective outreach, and engagement of the general public in taking action and changing behaviours? (Holly Arntzen)
250. **What are the spill-over effects in non-tropical MPAs and would MPAs contribute more to the ecosystem if they are no-take only?**
251. **Assuming the primary barrier identified for elected officials is "human values" which is the most effective means to change behavior with changes to human values? (Wayne Barchard)**
252. How can we reduce our human ecological footprint, when our governments are still managing as if the economy is separate from the ecosystem?
253. **How do we develop appropriate guidelines and techniques to restore the ecosystem after resource development has finished?**
254. **What stock market premium do firms that rate high in sustainability assessments command? [an update or further application of Derwall et al., “The Eco-Efficiency Premium Puzzle,” Financial Analysts Journal, vol. 61, no. 2 (2005), pp. 51-63].**
255. **What role will genetic technologies play in identifying, tracking, and monitoring rare species, species vulnerable to climate change, non-indigenous species, and cryptic species? (Steve Hill)**
256. ***What are the direct economic impacts of invasive species? [real values that $ can be assigned to, local level values important].***
257. ***To what extent do transboundary migrations affect or compromise Canadian efforts to manage, conserve or recover fish and animal species? (Steven Campana)***
258. Assuming the primary barrier identified for senior public servants is "management frameworks" and "organizational processes", which is the most effective means to change behavior with changes to human values? (Wayne Barchard)
259. **What are natural recovery times for marine and fresh-water ecosystems?**
260. What are the employment implications of a "green" economy?
261. How do we assign conservation value to non-charismatic organisms?
262. ***How can we develop generally accepted metrics for corporate initiatives in support of greater sustainability?***
263. **How does society provide alternative employment opportunities for those communities affected by conservation interventions?**
264. **How can society minimise political interference in conservation processes? How to increase bio-security protocols to limit non-native organisms entry into Canada?**
265. **How will climate change affect the distribution of non-native species in Canada, and their impacts on native species?**
266. **How do we change current legislation to ensure species at risk are listed in an appropriate timeframe?**
267. **How do we increase food production without further impairing natural ecosystem?**
268. **How do we assess the effectiveness of educational programmes on environmental issues? (Martin Willison)**
269. **Should we pursue assisted translocation for endangered species?**
270. **Where are the marine species and endemism hotspots in Canada?**
271. What is the optimal placement of marine PA in Canada and how can we remove the barriers to implementation of Marine PA?
272. **How do we make sure Canada upholds its global biodiversity and protection commitments?**
273. How do we put natural disturbance back on the landscape/seascape?
274. **How will climate change affect aquatic invasive species in the Arctic?**
275. What are some strategies for increasing public awareness and convincing communities to support protection initiatives?
276. How to educate society about impacts of non-native species on our environment?
277. How do we ensure the lack of funding for conservation research does not interfere with Canada's ability to provide scientific based information for legislation and policy?
278. How can we make sure there is sufficient funding for conservation research?
279. How do we assess the positive and negative affects of climate change on ecosystems?
280. **How do large-scale resource developments affect climate, pollution, habitat loss, [ecosystem services]?**
281. ***What are the cumulative genetic impacts of human activities (e.g., pollution, habitat disturbance) on animal and plant species?***
282. What role do contaminants and climate change play in the population decline of species at risk?
283. What is the impact of dams / reservoirs on greenhouse gas emissions?
284. ***To what extent can species at risk (large mammals, flora and fauna, species susceptible to disease) be protected and maintained by long-term parks and protected areas?***
285. **How can silviculture practices efficiently mitigate the impacts of climate change?**
286. How can habitat for aquatic fauna in Quebec be restored given the impacts of agriculture on watercourses throughout the province?
287. Which temporal and spatial factors need to be considered to predict structural and functional changes in ecosystems?
288. How do we persuade researchers to ask relevant questions which will be directly useful for politicians and administrators?
289. **How important is conservation to the general public?**
290. **How can we develop a Natural Capital Account System to identify areas of the country where we have overdrawn from our natural capital and to establish the carrying capacity on a (sub) ecosystem basis, to set growth limitation or reductions? (Lynn McIntyre)**
291. **How can systems of protected areas be better designed to protect biodiversity and ecosystem services under scenarios of a warming climate?**
292. What value are species, ecosystems, and ecological processes to the Canadians? [need to ask this at a sub population level - citizens, scientists, cultural groups, natural resource management workers, politicians, industry,..] (Rochelle Owen)
293. When will we realize that to manage Canada's Oceans we have to fully map the seafloor?
294. What is the most effective (in terms of preserving species diversity and ecosystem health) and logistically optimal method of conserving and restoring natural habitats in Canada? (Cassandra Silverio)
295. **What is the minimum habitat area and habitat quality required to sustain rare species in natural populations? (Jeremy Lundholm)**
296. ***How will climate change drivers, modelled at a regional scale, express themselves as weather locally, and how will local biotas respond? (Donald McLennan)***
297. **What land-use policies and best-practices need to be developed to ensure coastal ecosystems can adapt to rising sea-levels caused by climate change?**
298. How can quantification of the value of ecosystem services be used to inform policy related to economic growth and resource use? (Jeremy Lundholm)
299. **What are the net ecosystem services lost due to fishing activities in Canadian waters? (Susanna Fuller)**
300. What are the costs financially (i.e., government bailouts) and what are the true costs of restoring marine ecosystems degraded by fishing in Canada? (Susanna Fuller)
301. What is the most effective way of rebuilding and maintaining resilience in Canada's three oceans? (Susanna Fuller)
302. How best can we balance long-term ecosystem integrity and resilience with competing land, water and resource uses?
303. **How can we build a national inventory and monitoring system for crucial wetlands and their ecological services to support the conservation and sustainable management of these highly productive ecosystems in Canada? (Brian Kazmerik)**
304. What is causing the global decline in pollinators? (Claudia Haas)
305. How is Canada going to reduce carbon emissions in order to conserve the integrity of national and global ecosystems?
306. **What is the full range of impacts from salmon aquaculture operations, both directly to wild populations and to watershed ecosystems they support? How best can wild salmon be protected and fisheries be harvested with minimal harm? (Matt Stoecker)**
307. **What are the environmental impacts of offshore energy resources development (fossil fuel and renewable energy) and marine incidents?**
308. What are effective measures for sustainable natural resource development and marine pollution control?
309. ***What impact will resource development & exploration have in northern ecosystems (tundra, polar, etc) in a warming climate and what policies can be developed to mitigate extensive damage to these ecosystems? (Alex Zeller)***
310. **Can methods be proposed for valuing ecological benefits and time period be developed to evaluate the true cost and benefits of a given regulation or order under the RIAS process (e.g., SARA listing)? (Simon Nadeau)**
311. Which biomes or natural areas are experiencing the greatest threat due to human activities (industrial expansion, resource extraction, urbanization, etc)?
312. **What is the nature and value of ecosystem services provided by native prairie in western Canada and the location, quality, and area of native prairie parcels remaining in western Canada? (Dr. Rob Wright)**
313. **What are the practical conservation, social and economic objectives for ecosystem-based management in Canada? (Robert Stephenson)**
314. **What is the ecological literacy level of the average Canadian? (Ron Dodson)**
315. What is the value of our current ecosystem processes and systems? Should we develop over certain types of processes if we need to pay for them again, usually with public funds, in the long run?
316. **How does an individual's economic status shape his/her willingness to buy into conservation strategies and solutions to environmental problems?**
317. **Can a shift to a greener economy be used to improve the economic status of those that are marginalized and thus increase their "buy in"?**
318. **In the context of determining whether offshore MPAs are working, what are some credible indicators that can be used generally for groundfish, pelagics, etc. assuming species are migratory and fishing has occurred for years?**
319. How do we define conservation, differentiating between it and sustainable use?
320. **Which biodegradable plastics will cause least harm in cold terrestrial, aquatic and marine ecosystems if they become widely used for disposables packaging? [measures of harm include their persistence under various conditions, digestablility, bouyancy, toxicity and loss of strength over time]. (Laura Park)**
321. ***As Canada becomes more urban & with a higher proportion of the population not raised in Canada, attitudes to conservation may change. Will there be: greater use of resources (legal or illegal); less support for wildlife/wilderness protection; less tolerance of aboriginal, rural, traditional uses? (Heather Myers)***
322. **How effective have Canada's legal instruments - national and international, soft and hard law been at protecting our marine environment? Where are the legal and implementation gaps, and how should these gaps be filled (i.e., new policies or better governance)? (Susanna Fuller)**
323. **How will climate change impact current protected areas? How can protected areas be managed to minimize the impact of climate change? (Claudia Haas)**
324. **What governance changes are required to achieve effective Integrated Management (ecosystem-based management) in Canada? (Robert Stephenson)**
325. **What role should social and economic considerations play in identifying a species at risk to extinction?**
326. Can the "floating reserves" concept be applied in the boreal region as a realistic and practical strategy to complement biodiversity conservation in static protected areas, especially in the face of forest ecosystem dynamics at multiple spatial and temporal scales?
327. **Considering that habitat loss is the most significant threat to biodiversity, what are the quantifiable changes in the extent and distribution of various habitat types in Canada?**
328. **What is the best approach to engage landowners into actions to protect species at risk they have on their land?**
329. **What are the needed binding regulatory and legislative frameworks for EBM and sustainability of the oceans that ensure inclusion, coordination among, and clear authority/responsibility of federal, provincial, local, and First Nations governments?**
330. Does the vibro-acoustic profile of an industrial wind turbine pose a quantifiable health risk for those living in the vicinity of the development and, if so, how does risk vary with distance from the development? (Ruth Cooper)
331. **In light of the various sources of uncertainty in ecological and social systems, what management decisions/policies are more likely to yield favourable outcomes and less likely to yield catastrophic ones? (Robustness; can be applied to any system and any set of management/policy options) (Kai Chan)**
332. What can be done to reverse the erosion of fisheries management and research due to smaller budgets, reduced staffing and weaker laws under the Conservative government? (Jim Culp)
333. ***How can resource management and protected areas management be harmonized to realize goals for biodiversity conservation across a wider landscape (i.e., beyond protected areas boundaries)?***
334. **What are the real and modelled/anticipated large-scale and local impacts of climate change on Canada's oceans?**
335. ***What management strategies, frameworks, and institutions will help adapt to climate change impacts on Canada’s oceans?***
336. What are the cumulative impacts of human activities on ecosystem services? (Kai Chan)
337. What are the top 5 indicators that are easily monitored and tell us the trends of health in ocean ecosystems and how can we get management authorities to agree on and implement them?
338. What are the range of expected outcomes of actions A-D on ecosystem services (G-L) produced in ecosystem X? (Can be asked for any actions, ecosystem services, and ecosystem) (Kai Chan)
339. What are the range of expected implications of decisions/policies A-D on ecosystem services G-L in any ecosystem (local, remote, or global)? (Can be asked for any institution/organization and any set of decisions/policies and institutions/organizations.) (Kai Chan)
340. What is our build-out scenario for Canada's oceans? What mix of use and conservation do we want and need to ensure sustainable healthy oceans and communities?
341. How pivotal is both property assessing and retaining biodiversity in situ for human survival?
342. How do we identify spatial and temporal scales needed to address questions related to effective conservation and management of wildlife species, habitats and ecosystems?
343. **What incentives would facilitate collaboration between (academic) researchers and resource conservation managers?**
344. **How do we maintain national ecological monitoring programs under economic recessions?**
345. How can we develop meaningful ecological thresholds and report at a rate responsible to political expediency?
346. What is the impact of the mobile gear on the various coastal habitats?
347. **What would be the best social marketing approach to make protection of biodiversity a fundamental value for Canadians?**
348. **What is the best approach to lead people to protect species at risk on their private land?**
349. **What are the impacts of road construction, maintenance and salting on the persistence of plant and animal populations?**
350. What criteria will determine the possibility and viability of re-locating populations of plants?
351. What are the hallmarks of effective "critical habitat" for species at risk?
352. **What are the best means of protecting "critical habitat" for species at risk?**
353. What kind of public involvement makes for good conservation outcomes?
354. **Given climate change and the existing protected areas network, what are the best means of facilitating movement / connectivity of plants and animals?**
355. ***What ecological processes of global and/or continental significance are most strongly influenced by Canadian Federal Government legislation and policy?***
356. ***What are the real ecological impacts of human visitation to protected areas?***
357. **What are the most effective ways of managing uncertainty in environmental decision-making?**
358. To what extent and in which ways is human activity now the key driver of ecological and species evolution?
359. **What effect does the Atlantic Canadian seal population have on commercially important fish species?**
360. What must be done to more effectively engage public, stakeholder and government attention to addressing the significant impacts on marine ecosystem health that exist already, and to more expediently respond to them?
361. The seal hunt is a divisive issue in Canada. Should seals, even though they are not at risk, be considered a critical ecosystem service and, if so, what sort of financing / regulatory system can be used to enable conservationists to effectively buy out the commercial harvest?
362. How can Canada achieve an integrated approach to oceans stewardship that will effectively balance environmental imperatives and ocean use objectives, in a manner that recognizes that protecting ecosystem structure and function must be foremost in some particularly vulnerable places?
363. What science must be undertaken to more fully understand the likely impacts of climate change on marine ecosystems and biodiversity, and to plan their future uses and protection appropriately in both governance and societal terms?
364. **What processes and governance models would take us from species-based to an ecosystem-based approach for Canada's marine life? (Ishbel Munro)**
365. ***How will we pay to protect biodiversity when people don't want to raise taxes and there is such a demand on existing government funds? (Dennis O'Grady)***
366. How much and how soon do we need to find alternate energy reserves to avoid catastrophic change given dwindling oil supplies?
367. **What are consequences to biodiversity of climate-change induced hybridization?**
368. **How can endangered species legislation that protects critical habitat be applied to large-bodied species that inherently use a lot of space and thus conflict with human land use?**
369. Would it be beneficial to have a program that would encourage fish harvesters and perhaps give them some advantages to venture into aquaculture without fear of losing their livelihood? (Leslie-Anne Davidson)
370. **Can aquaculture be conducted in such a way to improve the ecosystem and endemic biodiversity of the culture site and surrounding areas? (Leslie-Anne Davidson)**
371. **How do we engage the federal government to leverage the provincial governments to manage species at risk? (SARA gives the mandate but the government has yet to exercise its authority to ensure good policy decisions at the provincial level) (Mark Boyce)**
372. **How can Canada best meet its international and ethical obligations towards combating climate change using land conservation tools? (Arne Mooers)**
373. **Which land management practices in agricultural systems can best support healthy wildlife populations and sustain farm families? (Glen Hvenegaard)**
374. What policies can we develop to manage watersheds holistically, incorporating cumulative effects of a wide variety of human activities? (John Reynolds)
375. How can we shift from a growth-bound paradigm to one that is sustainable? (Colin Soskolne)
376. How can society defend itself against self-interested leadership beyond annual reports to shareholders or election cycles? (Colin Soskolne)
377. **What will be the combined impacts of the commercialization of universities, increasing industrial partnerships by funding agencies, and continuing decreases in funding for basic ecological and evolutionary research on biodiversity and the environment?**
378. **Are Canadian/Provincial laws designed to protect species working (i.e. are species' numbers increasing)?**
379. Are amphibian relocations (often used as a mitigation measure during development and the Environmental Assessment process) successful?
380. Is the Environmental Assessment process (Federal or Provincial) effective at protecting species at risk?
381. Using an objective approach to assessing human values and value systems in relation to conservation progress, which specific values and value systems actually lead to sustained conservation gains, which ones lead to ephemeral gains, and which lead to conservation losses such as loss of biodiversity? (Martin Willison)
382. What is needed to restore fishery health (e.g. moratoriums on fishing in areas or certain species)? (Brennan Anstey)
383. What are the major threats to oceanic ecosystems and how can we monitor and reduce/eliminate impacts? (Brennan Anstey)
384. What harmful (or suspected harmful) chemicals are in use today and what are the replacement options and implications? (Brennan Anstey)
385. How can we make citizen more aware of the importance of healthy ecosystems in our everyday lives to improve quality of life? (Vivek Voora)
386. **What is the cumulative effect of pollutants and other stressors on water-based life (rivers, estuaries and marine)?**
387. How do we reconnect urban dwellers with the natural environment?
388. Should intensive densification of cities be favoured over rural and exurban development?
389. **What are the ranges and life history patterns of populations at risk (including data deficient populations), and how can Candians monitor and protect these systems?**
390. What governance changes are required to enable thresholds to be set by considering the limits and requirements/activities within a functional ecological system? (Ruth Waldick)
391. How can Canada better manage and integrate freshwater issues with questions of economic and community resilience, growth and increasing demand?
392. How can we better link the massive amounts of data and analysis concerning environmental change, human and population health, conservation efforts and management with both public opinion and public policy?
393. **What will be the burden (in health and economic terms) of environmental change on vulnerable communities and populations in Canada (the poor, women, racial minorities, rural communities and Aboriginal populations)?**
394. ***To what degree (empirically) can/do communtiy-based approaches toward natural resource management improve/contribute to the success of those approaches?***
395. **What are the social implications/dimensions of climate change and mitigation/adaptation strategies for rural communities in Canada?**
396. **How can we incorporate traditional indigenous knowledge in the development of adaptive policies addressing climate change in the boreal forest?**
